# Optimizing Feeding Regimens with Differential Protein Levels to Enhance Growth and Health in Juvenile Largemouth Bass (*Micropterus salmoides*)

**DOI:** 10.3390/ani16101542

**Published:** 2026-05-18

**Authors:** Yaling Xian, Fubao Wang, Zecheng Zou, Jiayi Wen, Yuping Chen, Jiping Zhang, Yongsheng Wang

**Affiliations:** 1 School of Animal Science and Technology, Foshan University, Foshan 528225, China; 2 Shenzhen Branch, Guangdong Laboratory of Lingnan Modern Agriculture, Agricultural Genomics Institute at Shenzhen, Chinese Academy of Agricultural Sciences, Shenzhen 518119, China; 3 Kunpeng Institute of Modern Agriculture at Foshan, Foshan 528225, China; 4 Guangdong Special Aquatic Functional Feed Engineering Technology Research Center, Guangdong Jieda Feed Co., Ltd., Foshan 528211, China

**Keywords:** *Micropterus salmoides*, growth performance, phased feeding, dietary protein level, immune markers, antioxidant capacity, intestinal microbiota

## Abstract

Largemouth bass (*Micropterus salmoides*) represents an important freshwater aquaculture species. Dietary protein strategies significantly influence growth performance, immune markers, and intestinal microbiota homeostasis. This study evaluated the physiological effects of five phased protein regimens on juvenile largemouth bass (9.77 ± 0.03 g) over 60 days. A constant 46% protein diet (CON) served as the control, compared with four dynamic regimens (LLH, LMH, LHH, HML) comprising low (L, 43%), medium (M, 46%), or high (H, 50%) protein levels across three 20-day phases. Seven hundred fish were randomly allocated into five groups (four replicates each). Daily feed intake was recorded, body weight was measured at the start and end, and survival rates were monitored. Post-trial assessments included body composition, serum biomarkers, intestinal morphology, and intestinal microbiota. Results showed that the LHH regimen significantly increased immunoglobulin M, complement C3 and C4, lysozyme activity, catalase, glutathione peroxidase, and total antioxidant capacity compared to the CON group. The LHH group also exhibited a reduced relative abundance of *Stenotrophomonas_A*. Functional predictions indicated downregulation of limonene/pinene degradation and the renin–angiotensin system. The LHH feeding regimen synergistically enhances immune-antioxidant function and optimizes intestinal microbiota in juvenile largemouth bass.

## Simple Summary

Feeding fish with the right amount of protein is essential for their growth and health, but finding the optimal feeding regimen remains a challenge for fish farmers. This study investigated the effects of dynamic protein feeding regimens on growth performance and health status in juvenile largemouth bass, an economically important aquaculture species. We tested five different protein feeding strategies with varying protein levels across different growth stages to determine which approach produces the healthiest and fastest-growing fish. Our results showed that adjusting protein levels at specific developmental stages significantly influenced fish metabolism and health outcomes. Fish fed with low protein at the start followed by high protein in the middle and final stages showed improved immune function and better antioxidant capacity in their blood. Those receiving low protein at the start and middle stages but high protein at the end showed enhanced digestive enzyme activity. The regimen of starting with low protein and maintaining high protein through the middle and final stages also promoted healthier intestinal bacteria diversity. This low–high–high protein approach proved most effective for balancing growth, immune health, antioxidant function, and intestinal microbiome stability. These findings provide practical feeding guidelines that enable fish farmers to raise healthier largemouth bass more efficiently, supporting sustainable and cost-effective freshwater aquaculture production.

## 1. Introduction

Global aquaculture production currently constitutes over 50% of the global edible fish supply, representing a critical component of food security [1,2]. However, the sustainable development of this industry faces dual challenges, including steadily increasing feed costs and growing environmental concerns [3,4]. As a high-value freshwater species, the largemouth bass (*Micropterus salmoides*) has experienced a rise in intensive farming due to its rapid growth rate and desirable meat quality [5,6]. Nevertheless, high-density aquaculture presents significant challenges in terms of cost management, fish health, and environmental sustainability [7].

In intensive aquaculture systems, feed costs typically exceed 50% of total production expenses. As the costliest component of feed, protein utilization efficiency directly influences economic viability [8]. Protein serves not only as a fundamental macronutrient for fish growth and development but also plays critical roles in various physiological processes, including tissue formation, energy metabolism, and immune function [9]. During the juvenile growth phase, protein intake is particularly decisive for growth performance and health status [10,11]. However, excessive dietary protein leads to increased ammonia nitrogen excretion via deamination, resulting in economic waste, water eutrophication, and metabolic stress [12]. Conventional fixed-protein diets often fail to accommodate the dynamic nutritional requirements of fish, leading to low utilization efficiency and environmental pollution. Therefore, optimizing protein utilization is essential for enhancing both the economic and environmental sustainability of aquaculture [13].

Dynamic protein feeding strategies, which adjust dietary protein levels according to developmental stages or environmental conditions, have gained increasing attention in juvenile fish culture. This approach has demonstrated efficacy in promoting growth, reducing costs, and mitigating nitrogen emissions. In juvenile fish, varying dietary protein levels through cyclic feeding regimes can optimize growth performance and feed efficiency. Research has shown that alternating feeding schedules with different protein levels improves growth and feed utilization in Nile tilapia through modulation of digestive enzyme activities and intestinal health [14]. Nutritional programming during early life stages has shown significant benefits. Feeding Nile tilapia fry restricted-protein diets followed by standard grow-out diets significantly improved growth and feed efficiency compared to continuous high-protein feeding [15]. Cyclic feed restriction and refeeding induce compensatory growth in juvenile Siberian sturgeon through mechanisms involving hyperphagia and improved feed conversion efficiency [16]. Similar strategies have produced favorable outcomes in juvenile milkfish under cyclic feeding regimes [17]. Juvenile rainbow trout exhibit compensatory growth after various levels of dietary protein restriction followed by refeeding [18]. Furthermore, juvenile matrinxã subjected to cyclic feed restriction achieved full compensatory growth with improved feed efficiency [19]. These findings demonstrate that strategic protein modulation during juvenile development enhances growth performance while reducing environmental impact and production costs.

Current research on largemouth bass nutrition has primarily focused on determining static protein requirements and evaluating alternative protein sources [20,21]. Studies on dynamic protein feeding regimes that cyclically modulate protein levels in this species remain limited. The optimal pattern, magnitude, and frequency of protein fluctuation that maximize utilization efficiency without compromising physiological health require further investigation. This study addresses this research need by designing five dynamic protein feeding regimens based on three common protein levels. The three dietary crude protein levels (43%, 46%, and 50%) were selected based on previously established protein requirement data for juvenile largemouth bass, for which optimal dietary crude protein has been reported in the range of 40–47% [22,23], with levels up to 50% commonly evaluated in controlled feeding trials for this species [24,25]. By systematically evaluating growth performance, body nutrient composition, immune parameters, and intestinal health, we aim to comprehensively assess the applicability of dynamic protein strategies for juvenile largemouth bass. The results will provide a theoretical foundation for developing efficient and environmentally sustainable aquaculture practices.

## 2. Materials and Methods

### 2.1. Animal Ethics

Institutional Review Board Statement: The use of juvenile largemouth bass in this study complied with the animal welfare laws, guidelines, and policies, as approved by the Scientific Ethics Committee of Kunpeng Institute of Modern Agriculture at Foshan, China (approval number: 20240303; approval date: 3 March 2024).

### 2.2. Experimental Design and Diets

**Figure 1:**
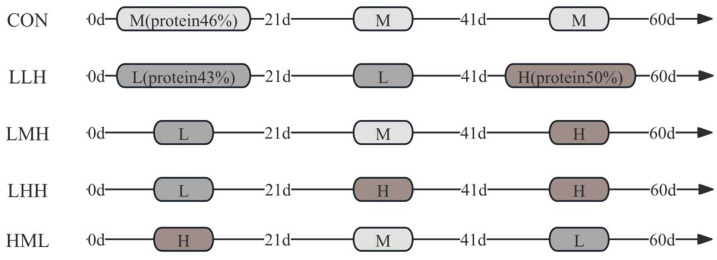
Schematic representation of differential protein-level feeding regimens across five experimental cohorts over 60 days. (L = Low-protein diet; M = Medium-protein diet; H = High-protein diet. CON, LLH, LMH, LHH, and HML represent different feeding regimens over 60 days divided into three phases of 20-day intervals. CON = sustained M-diet; LLH = L/L/H; LMH = L/M/H; LHH = L/H/H; HML = H/M/L).

Three isolipidic diets, designated L, M, and H, were formulated using fish meal and soybean meal as protein sources, fish oil and soybean oil as lipid sources, and starch as the carbohydrate source; they contained 43%, 46%, and 50% crude protein and had comparable gross energy (20.15, 20.17, and 20.29 MJ/kg DM, respectively; Table 1). The 60-day trial employed a phased feeding technique divided into three intervals: days 0–20, 21–40, and 41–60 (Figure 1). Five experimental groups were established: the CON group (control) adhered to the M-diet throughout the trial; the LLH group received the L-diet during days 0–20, L-diet during days 21–40, and H-diet during days 41–60; the LMH group received the L-diet during days 0–20, M-diet during days 21–40, and H-diet during days 41–60; the LHH group consumed the L-diet during days 0–20, H-diet during days 21–40, and H-diet during days 41–60; and the HML group was administered the H-diet during days 0–20, M-diet during days 21–40, and L-diet during days 41–60.

The juvenile largemouth bass were purchased from Heshi Aquatic Products Co. (Foshan, China), Ltd., and the experimental diets and facilities were supplied by Jieda Feed Co., Ltd. (Foshan, China). Following a two-week acclimation period with a basal diet, 700 healthy fish with an initial body weight of 9.77 ± 0.03 g were randomly distributed to 20 culture tanks. Fish were reared in a recirculating aquaculture system (RAS) comprising cylindrical fiberglass tanks (1 m diameter, 500 L total volume, 400 L working volume). The system operated on a 2 h exchange cycle and water was treated sequentially with a microscreen filter, a biofilter, and UV sterilization. Each tank was stocked with 35 fish and functioned as an independent replicate. The experiment consisted of five groups, each with four replicates. Throughout the 60-day feeding trial, fish were rigorously administered the designated diets following the phased feeding method at 08:00 and 18:00 daily. Feed consumption, death rates, and water quality metrics were documented: water temperature ranged from 27 to 30 °C, pH levels were between 7.6 and 7.9, ammonia nitrogen was ≤0.02 mg/L, nitrite was ≤0.2 mg/L, and dissolved oxygen was ≥6.0 mg/L.

### 2.3. Sample Collection

After a 24 h fasting interval on day 60 of the study, four fish from each replicate (16 per group) were randomly selected and anesthetized with 40 mg/L MS-222. Body weight and length were recorded before aseptic dissection of the liver, intestine, and intraperitoneal adipose tissue. Blood was extracted from the caudal vein of eight randomly selected fish per group and centrifuged at 3500 r/min for 15 min at 4 °C to obtain serum, which was subsequently kept at −80 °C for assessments of immune markers, antioxidant capacity, and enzyme activity. Simultaneously, intestinal samples were rapidly frozen in liquid nitrogen in sterile cryovials for microbiota analysis. Intestinal tissues from four additional fish per group were fixed in 4% paraformaldehyde for histological examination, and the remaining four fish per group were stored at −20 °C for body composition analysis.

### 2.4. Growth Performance and Body Indices Analysis

Data were collected from 80 samples across the five groups (*n* = 16). The initial body weight (*IBW*) and initial fish number (*N_i_*) were recorded on day 0 using an analytical balance with a precision of 0.01 g. Final body weight (*FBW*), number of dead (*N_d_*), and total feed intake (*TFI*) were documented on day 60. Uneaten feed was collected daily by siphoning 30 min after feeding, oven-dried at 60 °C, and weighed to calculate actual feed intake (*TFI* = feed supplied − uneaten feed). Body length (*L*), visceral weight (*W_v_*), and hepatic weight (*W_h_*) were assessed simultaneously during tissue sampling. Subsequent indices were computed based on the following parameters:

Survival rate (SR, %) = (*N_i_* − *N_d_*)/*N_i_* × 100%,

Feed intake rate (FIR, g g^−1^ d^−1^) = *TFI*/[((*IBW* + *FBW*)/2) × *d*],

Weight gain rate (WGR, %) = (*FBW* − *IBW*)/*IBW* × 100%,

Specific growth rate (SGR, %/d) = [ln (*FBW*) − ln (*IBW*)] × 100%/*d*,

Protein efficiency ratio (PER) = (*FBW − IBW*)/(*TFI* × *CP*)

Feed conversion ratio (FCR) = *TFI*/(*FBW − IBW*),

Condition factor (CF, g/cm^3^) = (*FBW*/*L*^3^) × 100%,

Viscerosomatic index (VSI, %) = (*W_v_*/*FBW)* × 100%,

Hepatosomatic index (HIS, %) = (*W_h_*/*FBW)* × 100%,

where *N_i_* is initial number of fish, *N_d_* is number of dead, *TFI* is the total feed intake (g), *FBW* is the final body weight (g), *IBW* is the initial body weight (g), *L* is the body length (cm), *W_v_* is the visceral weight (g), *W_h_* is the hepatic weight (g), and *d* is the number of experimental days (60 d), *CP* is the weighted average crude protein content (%) across the three feeding phases.

### 2.5. Body Nutrient Composition Analysis

Analysis of the nutrient composition of the body samples was performed according to AOAC standards [26]. The specimens were dried at 105 °C in a Memmert UF110 forced-air convection oven (Memmert, Schwabach, Germany) until a constant weight was obtained for moisture determination. The crude protein content was quantified using a Büchi K355/K437 Kjeldahl nitrogen analyzer (Büchi, Flawil, Switzerland). The crude fat content was measured using a Büchi E816 Soxhlet extractor (Büchi, Flawil, Switzerland). Ash content was determined after incineration at 550 °C for a minimum of 12 h.

### 2.6. Serum Immune Markers, Antioxidant Capacity, and Intestinal Enzyme Activity

The activities of catalase (CAT), glutathione peroxidase (GSH-Px), total antioxidant capacity (T-AOC), and malondialdehyde (MDA) were assessed using the Shanghai Yezhi UPLC-MS kit (Shanghai, China). The activities of intestinal trypsin, lipase, and α-amylase were quantitatively determined using commercial kits from Nanjing Jiancheng Bioengineering Institute (Nanjing, China), following the manufacturer’s instructions. Serum immunoglobulin M (IgM), complement components (C3/C4), and lysozyme (LZM) levels were quantified using kits from Nanjing Jiancheng Bioengineering Institute.

### 2.7. Analysis of Intestinal Histomorphology

Intestinal segments preserved in 4% paraformaldehyde were processed following standard histological procedures, including dehydration, clearing, paraffin embedding, and sectioning at 5 µm thickness using a rotary microtome (Leica RM2235, Leica Biosystems, Wetzlar, Germany). Sections were stained with hematoxylin and eosin (H&E) and mounted with neutral balsam. Target tissue regions were imaged at 50× magnification using Image-Pro Plus 6.0 software (Media Cybernetics, Rockville, MD, USA). Four fish per group (*n* = 4) were randomly selected from each of the five experimental groups. A single transverse section per fish was examined, and ten intact, vertically oriented villi were randomly selected and measured for villus height, villus width, and muscle thickness. Mean values per fish were calculated and used for statistical analysis.

### 2.8. DNA Extraction and High-Throughput Sequencing Analysis

Intestinal microbiota samples were collected by dissecting the intestinal tract and aseptically harvesting the luminal contents. The collected samples were immediately snap-frozen on dry ice, transported to PersonalBio (Shanghai, China), and stored at −80 °C until DNA extraction. Intestinal content samples from four fish per group (*n* = 4 per group, 20 samples in total) were used for 16S rRNA sequencing analysis. Extraction blanks and no-template PCR controls were included as negative controls to monitor contamination. Genomic DNA was isolated using the OMEGA Soil DNA Kit (D5635-02; Omega Bio-Tek, Norcross, GA, USA) according to the manufacturer’s instructions. DNA integrity was verified by 0.8% agarose gel electrophoresis, and concentration/purity was assessed using a NanoDrop One spectrophotometer (Thermo Fisher Scientific, Waltham, MA, USA). The V3–V4 hypervariable region of the bacterial 16S rRNA gene was amplified with the primers 338F (5′-ACTCCTACGGGAGGCAGCAG-3′) and 806R (5′-GGACTACHVGGGTWTCTAAT-3′), yielding an amplicon of approximately 468 bp. Purified amplicons were ligated with Illumina sequencing adapters to construct libraries, which were sequenced on the Illumina NovaSeq 6000 platform using 2 × 250 bp paired-end chemistry. The total read length (500 bp) fully covered the 468 bp target region, allowing sufficient overlap for reliable paired-end assembly. An average of 60,000 raw reads per sample was obtained. Raw paired-end reads were quality-filtered, denoised, and merged using the DADA2 (version 1.26.0) via the q2-dada2 plugin in QIIME2 (version 2023.7.0) with default parameters, generating amplicon sequence variants (ASVs) at 100% similarity. Taxonomic assignment of representative ASVs was performed against the SILVA 138.1 SSURef NR99 database, which was pre-trained for the V3–V4 region. Microbial community analyses were conducted at phylum and genus levels. Alpha diversity indicators, such as Chao1, Faith-pd, Shannon, Simpson, observed species, and Pielou_e indices, were calculated using QIIME2 (version 2023.7.0). Beta diversity was evaluated using Weighted_Unifrac dissimilarity, with principal coordinate analysis (PCoA) conducted in QIIME2 (version 2023.7.0) to visualize group differences. Putative metagenomic functions were predicted with PICRUSt2 (version 2.5.2) based on the KEGG Orthology (KO), Enzyme Commission (EC) numbers, and MetaCyc pathway databases. The Nearest Sequenced Taxon Index (NSTI) was calculated to assess prediction accuracy. Differential functional analysis was performed in R (version 4.3.2) using the clusterProfiler (version 4.10.0) and metagenomeSeq (version 1.40.0) packages.

### 2.9. Statistical Analysis

Statistical analyses were performed using SPSS 22.0 (IBM Corp., Armonk, NY, USA). Normality was assessed using the Shapiro–Wilk test. Parametric data were analyzed by one-way ANOVA followed by Duncan’s multiple range test. Non-parametric data were analyzed using the Kruskal–Wallis H test. When homogeneity of variance was violated (Levene’s test, *p* ≤ 0.05), Dunnett’s T3 test was applied for post hoc comparisons. Statistical significance was defined at *p* ≤ 0.05.

## 3. Results

### 3.1. Growth Performance, Body Indices, and Body Nutrient Composition Analysis

The LLH and HML groups had significantly lower final body weight (FBW), weight gain rate (WGR), and specific growth rate (SGR) in juvenile largemouth bass, whereas the HML group demonstrated a considerably elevated feed conversion ratio (FCR) along with a significantly lower protein efficiency ratio (PER) compared to the CON group (*p* ≤ 0.05; Table 2). Compared with the CON group, the survival rate (SR) in the LMH and LHH groups and the condition factor (CF) in the LMH group showed an increase; however, these differences were not statistically significant (*p* > 0.05). The crude protein (CP) content in fish bodies increased in all experimental groups compared to the CON group, although the crude lipid (CL) content decreased; nevertheless, neither change was statistically significant (*p* > 0.05; Table 2). Ash content exhibited no statistically significant variation across the groups (*p* > 0.05).

### 3.2. Serum Immune Markers

Compared to the CON group, the LHH and HML groups exhibited considerable elevation in the activity levels of LZM, C3, C4, and IgM, whereas the LLH and LMH groups demonstrated markedly reduced LZM activity relative to the CON group (*p* ˂ 0.001; Table 3). Subsequent analyses revealed that the C3 level in the LMH group was significantly lower than that in the CON group, and the C4 level in the LLH group also exhibited a statistically significant reduction (*p* ˂ 0.001).

### 3.3. Serum Antioxidant Capacity

Compared with the CON group, all experimental groups exhibited a significant increase in serum CAT activity in juvenile largemouth bass (*p* ˂ 0.001; Table 4). The LLH and LHH groups showed a substantial increase in T-AOC activity relative to the CON group (*p* ˂ 0.001). In contrast, the LMH and HML groups showed significant inhibitory effects (*p* ˂ 0.001). Furthermore, serum MDA levels in all experimental groups were markedly elevated compared with those in the CON group (*p* ˂ 0.001). The GSH-Px activity was considerably elevated in the LHH and HML groups (*p* ˂ 0.001). In contrast, it was significantly diminished in the LLH and LMH groups relative to that in the CON group (*p* ˂ 0.001).

### 3.4. Intestinal Enzyme Activity and Histomorphology

Experimental data indicated that various experimental groups significantly influenced the digestive enzyme activity of juvenile largemouth bass (*p* ˂ 0.001; Table 5). Trypsin activity in the LLH group was markedly higher than that in the CON group, whereas the other experimental groups exhibited a considerable reduction. Lipase activity was significantly higher in the CON group than in the other groups. The LMH group exhibited a significant increase in amylase activity compared with the CON group, whereas the other groups showed significant inhibitory effects (*p* ˂ 0.001).

**Figure 2:**
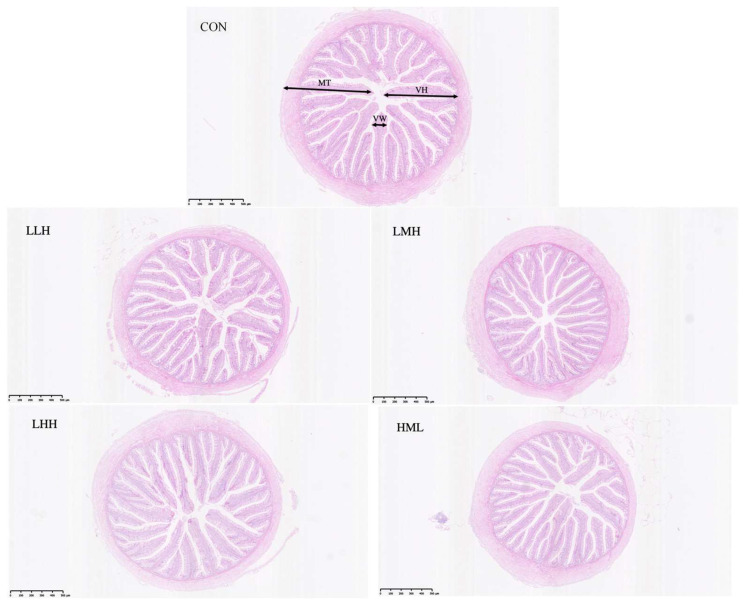
Effects of five feeding regimens at different protein levels on the intestinal morphologic structure of juvenile largemouth bass (*Micropterus salmoides*). (VH: Villus height; VW: Villus width; MT: Muscle thickness; L = Low-protein diet; M = Medium-protein diet; H = High-protein diet. CON, LLH, LMH, LHH, and HML represent different feeding regimens over 60 days divided into three phases of 20-day intervals. CON = sustained M-diet; LLH = L/L/H; LMH = L/M/H; LHH = L/H/H; HML = H/M/L).

All experimental groups exhibited an increase in villus height relative to the CON group; however, the differences were not statistically significant (*p* > 0.05; Table 6; Figure 2). No statistically significant differences were observed between the experimental groups in terms of villus width or muscle layer thickness (*p* > 0.05).

### 3.5. Alpha Diversity and Taxonomic Composition of Intestinal Microbiota

**Figure 3:**
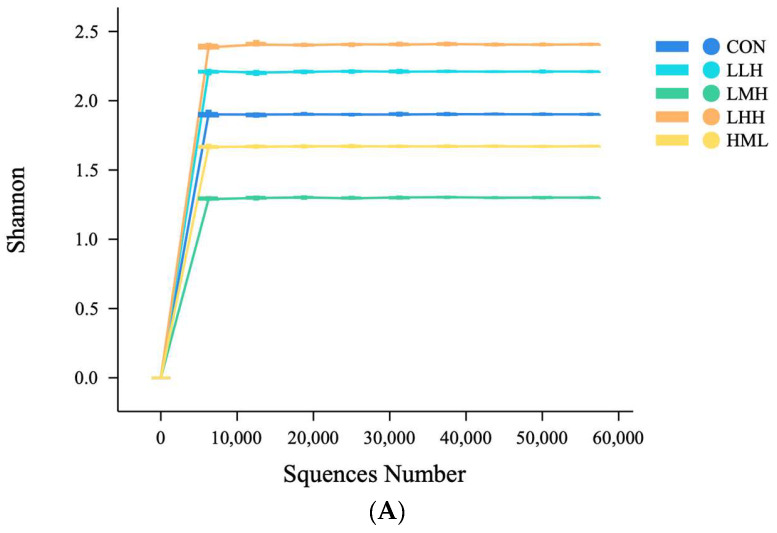

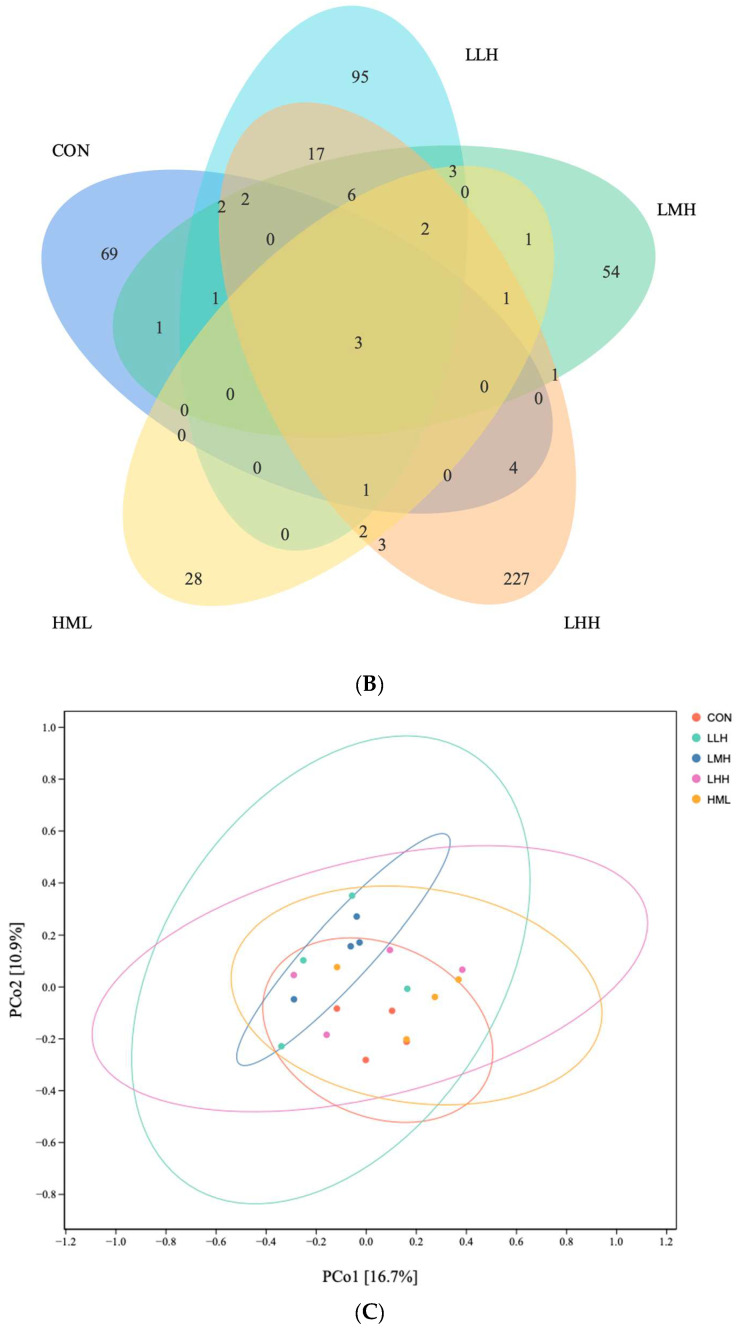
(**A**) Rarefaction curves of observed amplicon sequence variants (ASVs) in each group; (**B**) Venn diagram showing the number of microbial ASV shared within and between groups of samples; (**C**) Principal coordinate analysis (PCoA) showing β-diversity of cecal microbiota among groups. (L = Low-protein diet; M = Medium-protein diet; H = High-protein diet. CON, LLH, LMH, LHH, and HML represent different feeding regimens over 60 days divided into three phases of 20-day intervals. CON = sustained M-diet; LLH = L/L/H; LMH = L/M/H; LHH = L/H/H; HML = H/M/L).

The flattening of the sequencing data dilution curve indicated an adequate sequencing depth (Figure 3A). Following quality control, a total of 523 unique amplicon sequence variants (ASVs) were obtained. The number of ASVs unique to the CON, LLH, LMH, LHH, and HML groups was 69, 95, 54, 227, and 28, respectively. Additionally, the five groups shared three core ASVs (Figure 3B). Alpha diversity analysis indicated that none of the evaluated indices differed significantly among groups. (*p* > 0.05; Table 7). Similarly, PCoA based on β-diversity showed considerable overlap among groups, suggesting no marked effect of dietary protein levels on the cecal microbiota structure (Figure 3C).

**Figure 4:**
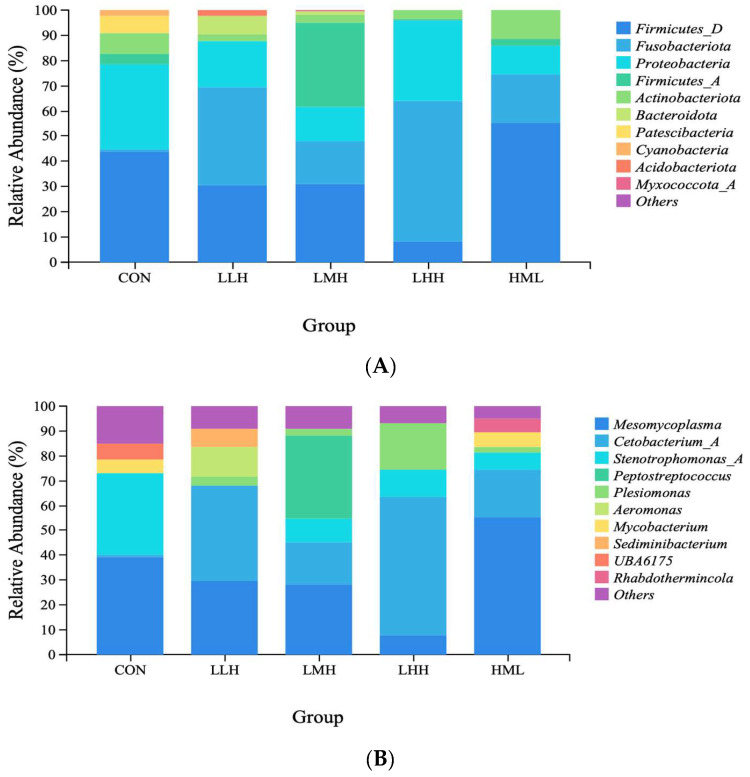
(**A**) Phylum-level taxonomic composition of the intestinal microbiota communities; (**B**) Genus-level taxonomic composition of the intestinal microbiota communities. (L = Low-protein diet; M = Medium-protein diet; H = High-protein diet. CON, LLH, LMH, LHH, and HML represent different feeding regimens over 60 days divided into three phases of 20-day intervals. CON = sustained M-diet; LLH = L/L/H; LMH = L/M/H; LHH = L/H/H; HML = H/M/L).

The top 10 dominant microbiota groups in the phylum region were *Firmicutes_D*, *Fusobacteriota*, *Proteobacteria, Firmicutes_A*, *Actinobacteriota*, *Bacteroidota*, *Patescibacteria*, *Cyanobacteria*, *Acidobacteriota*, and *Myxococcota_A* (Figure 4A). The top 10 dominant groups in the genus region were *Mesomycoplasma*, *Cetobacterium_A*, *Stenotrophomonas*, *Peptostreptococcus*, *Plesiomonas*, *Aeromonas*, *Mycobacterium*, *Sediminibacterium, UBA617*, and *Rhabdothermincola* (Figure 4B).

The horizontal community structure of the phylum indicated that the CON group was predominantly composed of *Proteobacteria* and *Firmicutes*; *Fusobacteriota* and *Firmicutes_D* primarily characterized the LLH group; the LMH group was enriched with *Firmicutes_A* and *Firmicutes_D*; and the LHH group displayed a synergistic predominance of *Fusobacteriota* and *Proteobacteria*. The HML group was predominantly composed of *Firmicutes_D* and *Fusobacteriota*. The relative abundance of *Firmicutes_D* in the LHH group was lower than that in the other groups (*p* > 0.05). Genus-level studies indicated that the five predominant genera across all groups were *Mesomycoplasma*, *Cetobacterium_A*, *Stenotrophomonas_A*, *Peptostreptococcus*, and *Plesiomonas*; however, variations in abundance within groups were observed. The relative abundance of *Cetobacterium_A* was lower in the CON group than in the experimental groups. The abundance of *Stenotrophomonas_A* in the LMH, LHH, and HML groups was more than three times lower than that in the CON group. This genus was not detected in the LLH group (<0.1%). *Peptostreptococcus* was notably enriched in the LMH group with an abundance below 0.1% in the other groups.

### 3.6. Microbial Function Prediction

**Figure 5:**
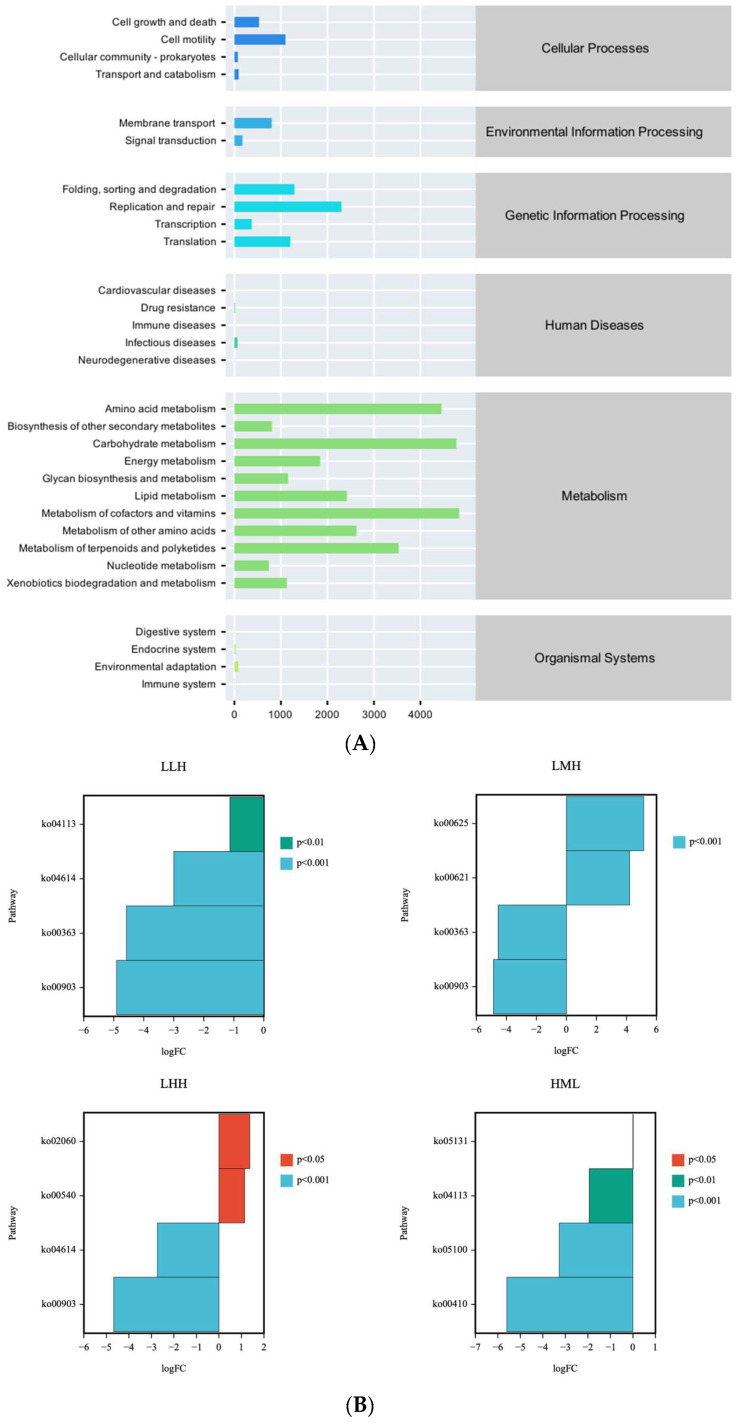
Function predictions using PICRUSt2. (**A**) Comparative analysis of biological processes and systems counts across different categories; (**B**) Differential metabolic pathway abundance across microbiome groups (L = Low-protein diet; M = Medium-protein diet; H = High-protein diet. CON, LLH, LMH, LHH, and HML represent different feeding regimens over 60 days divided into three phases of 20-day intervals. CON = sustained M-diet; LLH = L/L/H; LMH = L/M/H; LHH = L/H/H; HML = H/M/L).

PICRUSt2 analysis showed differences in predicted microbial functions between groups (Figure 5). KEGG metabolic pathway analysis showed that, at the primary functional level, the pathways with the highest abundance of microbial functional genes across all samples were Metabolism and Genetic Information Processing.

At the secondary functional level, 11 predominant pathways were identified within amino acid metabolism, carbohydrate metabolism, and the metabolism of cofactors and vitamins. These included: biosynthesis of other secondary metabolites, energy metabolism, glycan biosynthesis and metabolism, lipid metabolism, metabolism of different amino acids, metabolism of terpenoids and polyketides, nucleotide metabolism, and xenobiotic biodegradation and metabolism.

The KEGG metabolic pathway differential analysis (Figure 5) revealed distinct functional gene expression patterns in each experimental group relative to the CON group. The LLH group exhibited notable downregulation of the ko04113 (meiosis–yeast, *p* < 0.01), ko04614 (renin–angiotensin system, RAS, *p* < 0.001), ko00363 (bisphenol degradation, *p* < 0.001), and ko00903 (limonene and pinene degradation, *p* < 0.001) pathways. The LMH group markedly upregulated the ko00625 (chloroalkane and chloroalkene degradation, *p* < 0.001) and ko00621 (dioxin degradation, *p* < 0.001) pathways and downregulated the ko00363 (*p* < 0.001) and ko00903 (*p* < 0.001) pathways. In the LHH group, the ko02060 (phosphatase transfer system, *p* ≤ 0.05) and ko00540 (lipopolysaccharide biosynthesis, *p* ≤ 0.05) pathways were significantly upregulated, whereas the ko04614 (*p* < 0.001) and ko00903 (*p* < 0.001) pathways were downregulated. In the HML group, the ko04113 (*p* < 0.01), ko05100 (bacterial invasion of epithelial cells, *p* < 0.001), and ko00410 (beta-alanine metabolism, *p* < 0.001) pathways were significantly downregulated.

## 4. Discussion

### 4.1. Growth Performance, Body Indices, and Body Nutrient Composition Analysis

In ichthyological nutrition research, the SGR and WGR are fundamental metrics for assessing growth performance, whereas the FCR indicates feed utilization efficiency [27]. The SGR and WGR of juvenile largemouth bass fed LMH and LHH protein levels did not differ from the CON group. Prior research indicates that WGR and SGR increase with elevated feed protein levels and stabilize upon reaching a plateau [28,29,30]. Anderson et al. [22] determined minimum protein requirements for juvenile largemouth bass, indicating that protein levels above this threshold do not necessarily enhance growth. In the LMH and LHH feeding regimens, variations in feed protein levels did not influence growth performance, probably because the protein levels approached the plateau necessary for optimal fish growth. No significant differences in FCR were detected among the CON, LMH, LLH, and LHH groups (*p* > 0.05). By contrast, the FCR of the LHH group was significantly lower than that of the HML group (*p* < 0.05). This trend likely reflects the cumulative effect of continuous high-protein feeding in the final two stages, as adequate protein levels and balanced amino acids are known to improve growth efficiency and reduce FCR [31]. The reduced PER in the HML group indicates impaired protein utilization efficiency, consistent with the elevated FCR observed in this treatment [32]. Suboptimal dietary protein forces catabolism of amino acids for energy rather than protein deposition, thereby lowering PER [33].

Fish growth is accompanied by protein synthesis and deposition, and juvenile fish need to break down proteins into amino acids to support rapid growth and metabolic processes [34]. Li et al. [24] demonstrated that dietary protein and lipid levels interact to influence growth performance and protein utilization in largemouth bass, with optimal dietary crude protein and lipid levels supporting efficient protein deposition. When feed lipids and total energy were held approximately constant, fish in the experimental group tended to show higher crude protein content than those in the CON group. This aligns with observations that adequate dietary lipids spare protein from oxidative catabolism by serving as the primary energy substrate, thereby promoting protein deposition [35,36]. Lu et al. [37] showed that Lysphospholipid supplementation in low protein or low lipid diets enhances hepatic lipid metabolism and promotes protein deposition in largemouth bass, confirming the protein-sparing effect of lipids when dietary energy is maintained at similar levels. The decreased body fat content in the experimental group was attributed to alterations in feed protein content, which heightened metabolic regulatory energy expenditure, thus diminishing fat deposition efficiency [23].

### 4.2. Serum Immune Markers

The regulation of protein expression and function can dramatically impact the immunological response of fish, thereby improving their resistance to pathogens [38]. Lysozyme exerts antibacterial effects by lysing cell walls of harmful bacteria and serves as a significant element of the innate immune system in fish [39,40]. The complement system functions as a fundamental defense mechanism against microbial infections, facilitated by essential components including C3 and C4 [41]. Immunoglobulins are the initial specific antibodies generated by the body following antigenic stimulation [42,43]. The context-dependent nature of immune priming suggests that the persistence of elevated immune markers may vary with host genotype and environmental conditions [44,45]. Dietary proteins serve as immunoregulatory agents that promote immune protein synthesis [46]. Moreover, they influence host immunological responses by modifying the composition of the intestinal microbiota, selectively activating immune pathways, including the complement system, and regulating stress levels to enhance immune function [47,48]. This study revealed that IgM, complement components (C3 and C4), and lysozyme activity in the serum of largemouth bass fry in the LHH and HML groups were considerably elevated compared to those in the CON group. The HML group maintained elevated immune markers despite late-stage protein restriction. This pattern is consistent with immune priming effects, where prior nutritional exposure can enhance subsequent immune responsiveness [44,45]. However, sustained low protein likely impairs de novo synthesis of immune proteins, potentially limiting long-term competence despite transient marker elevation [49]. In contrast, the LHH pattern provided sustained high-protein availability, supporting continuous synthesis of immunoglobulins and complement proteins while avoiding scarcity-associated metabolic stress [50]. This reveals that the LHH group is superior to a singular protein-feeding regimen and can more effectively fulfill the fish’s immune system requirements.

### 4.3. Serum Antioxidant Capacity

Antioxidant defense mechanisms in fish comprise enzymatic and non-enzymatic systems. CAT efficiently scavenges reactive oxygen species including superoxide anion radicals, hydroxyl radicals, and hydrogen peroxide [51]. GSH-Px catalyzes the reduction of hydrogen peroxide and lipid hydroperoxides to water and corresponding alcohols, utilizing glutathione as a reducing agent [52]. T-AOC denotes the overall antioxidant capacity of macromolecules, small molecules, and enzyme-mediated systems [53]. MDA serves as a biomarker of lipid peroxidation, indicating oxidative damage to cell membranes caused by reactive oxygen species attack on polyunsaturated fatty acids [54].

The feeding regimen of the LMH and LHH groups significantly elevated CAT and T-AOC activities in serum. This may be ascribed to elevated protein levels resulting in heightened metabolic strain in the liver, stimulating augmented generation of reactive oxygen species, thus triggering a compensatory rise in CAT levels [55,56]. GSH-Px activity was also elevated in the LHH group, suggesting that sustained high-protein availability supports both enzyme synthesis and glutathione substrate provision [56]. The elevated protein levels in the LHH group may have activated CAT, GSH-Px, and other non-enzymatic antioxidant mechanisms, thereby augmenting T-AOC activity [57]. Compared with the CON group, the HML group exhibited elevated activities of CAT and GSH-Px but reduced T-AOC. This dissociation arose because graded protein changes induced compensatory enzyme upregulation via the Nrf2 pathway, but the subsequent low-protein phase limited substrates for glutathione synthesis [58,59]. Nrf2 is a key regulator of antioxidant responses that protects against oxidative stress by modulating antioxidant enzyme expression [60,61]. Nrf2 activation by amino acids enhances antioxidant enzyme expression and reduces oxidative stress in fish, highlighting the link between nutritional status and antioxidant capacity [60,62]. Consequently, abundant but substrate-limited GSH-Px consumed GSH and produced GSSG, while impaired GSH regeneration diminished the GSH/GSSG ratio [59]. This compromised system capacity and reduced T-AOC [60]. CAT-mediated H_2_O_2_ dismutation requires no reductants, thus remaining active [60]. The LLH and LMH groups exhibited reduced GSH-Px activity, indicating that early low-protein exposure or sustained moderate–high protein followed by restriction may limit glutathione substrate availability despite compensatory mechanisms [56]. All experimental groups exhibited a concurrent increase in both compensatory antioxidant enzyme activities and oxidative stress, the latter indicated by elevated MDA levels. This paradox is attributed to asynchronous amino acid supply resulting from dynamic dietary protein changes, a process that disrupts metabolic homeostasis, induces mitochondrial electron leakage, and ultimately leads to reactive oxygen species generation that exceeds the instantaneous antioxidant capacity [63,64].

### 4.4. Intestinal Enzyme Activity and Histomorphology

Digestive enzyme activity is crucial for evaluating digestive function in fish and is governed by dietary nutrient content [65,66].The intestinal trypsin activity of juvenile largemouth bass in the LLH group was significantly elevated compared to other groups, indicating superior digestive capabilities. The significantly elevated trypsin activity in the LLH group likely reflects a compensatory upregulation of protease secretion in response to the abrupt dietary protein transition in the final phase. Digestive enzyme activities in fish are known to respond dynamically to changes in dietary protein content [65,66], and alternating feeding regimes involving a protein-restricted period followed by high-protein realimentation have been shown to induce compensatory changes in intestinal enzyme secretion in teleost fish [14,15]. The CON group exhibited a significant increase in lipase activity and crude fat content within body composition, indicating that enhanced fat deposition may stimulate lipase secretion via a positive feedback mechanism [67]. Moreover, high-protein feed may indirectly augment lipase secretion capacity by modifying the intestinal microbiota [68]. Therefore, periodically adjusting the protein level in feed can effectively mitigate excessive fat deposition and prevent digestive enzyme activity disorders.

The intestine is the primary organ for digestion and nutrient absorption in fish, and its normal shape is essential for sustaining functional homeostasis [69]. Histological analysis indicated no significant alterations in the height and width of the villi or muscle thickness of juvenile largemouth bass across experimental groups. Similar phenomena have been documented in studies of cyprinid fish. This result may stem from the limited variation in protein levels in the feed, which did not attain the threshold necessary to influence the intestinal structure [70]. This indicated that feeding regimen with phased adjustments to protein levels did not adversely affect the intestinal morphology of juvenile largemouth bass.

### 4.5. Alpha Diversity and Taxonomic Composition of Intestinal Microbiota

The intestinal microbiota of fish serves as a key regulator of host nutrient digestion, immune response, intestinal development, and disease resistance [71]. Its composition is closely linked to fish species, developmental stage, diet composition, and rearing environment [72]. Dietary components influence host physiological function by altering intestinal microbiota structure [73]. In the present study, no significant differences were found in various alpha diversity indices among all experimental groups, and PCoA based on β-diversity also revealed considerable overlap among groups, which may indicate that dietary protein levels have a limited effect on the diversity and overall structure of the gut microbiota in juvenile largemouth bass [74]. It should also be noted that factors such as sample size and interindividual variation may have influenced the results to some extent. At the phylum level, *Fusobacteriota* emerged as the dominant microbiota in the LLH, LHH, and HML groups relative to the CON group. This phenomenon may be attributed to the role of *Fusobacteriota* in mediating protein degradation in fish intestines [75]. The LLH, LHH, and HML feeding regimens all involved high-protein diets, which likely resulted in an increased abundance of this microbiota. *Firmicutes_D* abundance was significantly decreased in the LLH, LMH, and LHH groups compared to the CON group, reflecting intestinal microbiota optimization during the late high-protein stage. Previous studies reported that 46.28%~51.0% dietary protein promotes proliferation of functionally defined genera (*Clostridium* and *Lactococcus*), compresses the niche of unclassified *Firmicutes*, and reduces opportunistic pathogens (*Staphylococcus*), indicating that *Firmicutes_D* reduction signifies functional maturation of the intestinal microecosystem [25]. At the genus level, the relative abundance of *Cetobacterium_A* was lower in the CON group than in the other groups. This genus is essential for vitamin B_12_ production and modulates protein and lipid metabolism [76,77]. Furthermore, its metabolites markedly improve the efficiency of dietary nutrient utilization in fish by influencing the intestinal microbiota and metabolic pathways [78,79]. In addition, *Stenotrophomonas_A* was more abundant in the CON group than in the other groups. This pathogenic bacterium typically induces infection when the immune system is compromised, whereas in the intestines of healthy individuals, symbiotic flora (such as lactic acid bacteria that produce hydrogen peroxide and bacteriocin-like substances) can suppress their proliferation [80]. The relative abundance of *Peptostreptococcus* in the LMH group was substantially higher than that in the other groups. This genus produces short-chain fatty acids (SCFAs) such as propionic acid and butyric acid by decomposing proteins in the mammalian intestine, providing energy to the intestinal epithelium, and inhibiting pathogenic bacteria [81]. Although there are differences between aquatic and mammalian intestinal environments, *Peptostreptococcus* may retain similar metabolic functions [82].

### 4.6. Microbial Function Prediction

KEGG functional prediction analysis based on 16S rRNA sequencing revealed that the predominantly enriched functional categories were amino acid metabolism, carbohydrate metabolism, and the metabolism of cofactors and vitamins. Amino acid metabolism facilitates nutrient absorption and transport by breaking down undigested dietary proteins and amino acids [83]. Additionally, this metabolic system regulates immunological responses and oxidative stress defense, while facilitating protein synthesis and cellular energy metabolism [84,85,86]. The low efficiency of carbohydrate consumption in fish leads to the breakdown of complex carbohydrates by intestinal microbiota, resulting in the production of SCFAs that supply energy to the host and maintain blood glucose homeostasis [87]. Carbohydrate and lipid metabolism are cooperatively regulated through gut microbiota-derived SCFAs, which modulate host energy homeostasis by activating PPARγ to enhance GLUT4-mediated glucose transport and attenuate lipid-associated inflammatory responses [88,89]. The metabolism of cofactors and vitamins supports host energy metabolism and detoxification via synthesis of bioactive molecules. For instance, glutamine precursors such as α-ketoglutaric acid enhance nutrient absorption and utilization through activation of intestinal alkaline phosphatase (AKP) [90].

Using 16S rRNA sequencing combined with KEGG functional prediction, this study demonstrated that dietary protein level significantly influenced the metabolic functions of the intestinal microbiota in juvenile largemouth bass. Specifically, the activity of pathway ko00903 (limonene and pinene degradation) was significantly lower in the LLH, LMH, and LHH groups compared with the CON group. This suppression may benefit host health through two complementary mechanisms. First, it promotes the accumulation of plant-derived monoterpenoid compounds with antibacterial and anti-inflammatory properties within the intestinal lumen, thereby limiting the proliferation of opportunistic pathogens within the intestinal lumen through disruption of bacterial membrane integrity and inhibition of virulence factor expression [91,92]. Second, reduced monoterpene degradation may enhance intestinal antioxidant capacity through activation of the Nrf2/HO-1 cytoprotective signaling pathway, thereby attenuating oxidative stress induced by high-fat diets and supporting intestinal barrier integrity [58,60]. In the LHH group, pathways ko02060 (phosphotransferase system, PTS) and ko00540 (lipopolysaccharide biosynthesis) were significantly upregulated, suggesting that this feeding regimen enhanced microbial glycolytic capacity through activation of the PTS [93]. Furthermore, the downregulation of pathway ko04614 (renin-angiotensin system, RAS) in the LHH group may alleviate intestinal and hepatic oxidative damage by reducing Angiotensin II (Ang II)-mediated NADPH oxidase activity and suppressing the NF-κB inflammatory signaling pathway [94,95], a mechanism consistent with the emerging link between gut microbiota composition and RAS modulation [96]. In the HML group, downregulation of pathway ko05100 (bacterial invasion of epithelial cells), its suppression may reflect enhanced host defense through probiotic-driven competitive exclusion of pathogens and bacteriocin secretion, alongside upregulation of mucus layer-associated gene expression that reinforces the physical intestinal barrier [97,98,99,100]. It should be noted that these functional pathway analyses are based on microbial community predictions rather than direct experimental validation, and further mechanistic studies are warranted to confirm these findings.

While the present study provides novel insights into the effects of phased dietary protein regimens on growth performance, immune function, and intestinal microbiota homeostasis in juvenile largemouth bass, several methodological considerations merit acknowledgment. The KEGG functional pathway analyses were derived from PICRUSt2-based computational predictions using 16S rRNA amplicon sequencing data, which, as is inherent to this widely adopted approach in aquaculture microbiome research, reflect inferred metabolic potential based on taxonomic composition rather than direct measurements of microbial gene expression or enzymatic activity. These predictions are therefore best interpreted as hypothesis-generating frameworks, and their biological relevance would benefit from further validation through complementary metagenomic or metabolomic profiling in subsequent studies. Furthermore, as is characteristic of observational microbiome research, the associations identified between dietary protein regimens, intestinal microbial community structure, and host physiological parameters are correlational in nature. Targeted interventional approaches, such as gnotobiotic animal models or microbiota transplantation experiments, would contribute to elucidating the precise causal mechanisms underlying the diet-microbiota-host interactions reported here.

## 5. Conclusions

In conclusion, this study elucidated the regulatory roles of stage-specific dietary protein adjustments on growth performance, immune function, antioxidant capacity, and intestinal microbiota homeostasis in juvenile largemouth bass. Among the five feeding strategies evaluated, the LHH and LMH feeding strategies sustained growth performance comparable to that of the constant-protein control, while the LHH group additionally demonstrated marked improvements in serum immune markers and antioxidant enzyme activities. The LLH feeding regimen further elicited a compensatory elevation in intestinal trypsin activity, reflecting an adaptive digestive response to the phased dietary protein transition. Collectively, the present findings indicate that the LHH phased protein feeding regimen holds considerable promise as a practically applicable approach for simultaneously optimizing immune competence and antioxidant defense in intensively cultured juvenile largemouth bass. Additionally, this regimen was associated with a reduction in the abundance of a potentially opportunistic genus (*Stenotrophomonas_A*), without significantly altering overall intestinal microbiota diversity or structure. Future studies incorporating metagenomics and metabolomics are warranted to validate the predicted functional pathways and further elucidate the mechanisms underlying the observed microbiota–host interactions under dynamic protein feeding strategies.

## Figures and Tables

**Table 1:** Experimental diet composition and nutrients (as-fed basis, %).

Item	L	M	H
Ingredient Composition (%)			
Fish meal	20.00	30.00	40.00
Soybean meal	24.80	17.80	8.90
Chicken meal	10.00	10.00	10.00
Soybean oil	6.50	6.60	6.70
Fish oil	2.00	1.00	
Soybean protein concentrate	10.00	10.00	10.00
Cottonseed protein concentrate	8.00	8.00	8.00
Spray-dried animal blood cells			2.00
Tapioca	12.00	11.00	10.00
Yeast extract paste	2.00	2.00	2.00
Ca (H_2_PO_4_)_2_	2.00	1.50	1.00
Carnivorous fish premix	1.00	1.00	1.00
Choline	0.40	0.40	0.40
Lysine (98.5%)	1.00	0.60	
Methionine	0.30	0.10	
	100.00	100.00	100.00
Calculated Nutrient Level (g 100 g^−1^ DM)			
Crude protein	43.30	46.20	50.00
Crude lipid	12.00	12.00	12.00
Crude ash	10.70	11.50	12.50
Lysine	3.39	3.39	3.39
Methionine	1.12	1.12	1.12
Threonine	2.06	2.06	2.06
Tryptophan	0.56	0.56	0.56
Leucine	3.46	3.46	3.46
Isoleucine	2.02	2.02	2.02
Valine	2.40	2.40	2.40
Phenylalanine	2.10	2.10	2.10
Histidine	1.29	1.29	1.29
Arginine	3.00	3.00	3.00
Gross energy (MJ/kg DM)	20.15	20.17	20.29

Note: L = Low-protein diet; M = Medium-protein diet; H = High-protein diet; DM = Dry matter. Vitamin premix provided the following per kilogram diet: vitamin B_1_, 20 mg; riboflavin, 20 mg; vitamin B_6_, 12 mg; vitamin B_12_, 0. 15 mg; vitamin K_3_, 12 mg; inositol, 300 mg; pantothenic acid, 60 mg; niacin acid 70 mg, folic acid 10 mg, biotin 1 mg, vitamin A 8000 IU, vitamin D_3_ 2000 IU, vitamin E 100 mg, vitamin C 500 mg, ethoxyquin 200 mg, defatted rice bran 535 mg. Mineral premix provided the following per kilogram diet: KCl 250 mg, KI (1%) 80 mg, CoCl_2_•6H_2_O (1%) 70 mg, CuSO_4_•5H_2_O 40 mg, FeSO_4_•H_2_O 500 mg, ZnSO_4_•H_2_O 500 mg, MnSO_4_•H_2_O 200 mg, Na_2_SeO_3_•5H_2_O (1%) 70 mg, MgSO_4_•H_2_O 2500 mg, zeolite power 3790 mg.

**Table 2:** Effects of five feeding regimens at differential protein levels on growth performance, body indices, and body nutrient composition of juvenile largemouth bass (*Micropterus salmoides*).

Item	CON	LLH	LMH	LHH	HML	SEM	*p*-Value
Growth performance							
IBW (g)	9.78	9.76	9.77	9.78	9.79	0.017	0.302
FBW (g)	77.61 ^a^	74.46 ^bc^	76.19 ^ab^	76.57 ^ab^	73.72 ^c^	1.099	0.018
WGR (%)	689.25 ^a^	661.65 ^bc^	679.73 ^ab^	683 ^ab^	649.85 ^c^	10.396	0.009
SGR (%/d)	3.44 ^a^	3.37 ^b^	3.42 ^a^	3.43 ^a^	3.35 ^b^	0.023	0.006
FIR (g g^−1^ d^−1^)	57.11	55.26	56.21	55.98	55.34	0.983	0.364
SR (%)	99.29	99.29	100.00	100.00	99.29	0.783	0.736
PER	2.55 ^ab^	2.56 ^a^	2.56 ^a^	2.50 ^b^	2.45 ^c^	0.02	0.001
FCR	0.85 ^b^	0.85 ^b^	0.85 ^b^	0.84 ^b^	0.87 ^a^	0.007	0.006
Body indices							
CF (g/cm^3^)	2.05	2.08	2.18	2.04	2.03	0.063	0.129
HIS (%)	8.15	8.00	7.70	7.81	8.00	0.378	0.785
VSI (%)	2.89	2.85	2.61	2.71	2.97	0.293	0.749
Body nutrient composition							
CP (%)	17.77	18.40	18.50	18.30	18.07	0.517	0.968
CL (%)	6.58	5.55	5.10	6.08	5.28	0.597	0.137
Ash (%)	3.47	3.23	3.27	3.30	3.40	0.101	0.417

Note: L = Low-protein diet; M = Medium-protein diet; H = High-protein diet. a–c Values in the same row with different superscripts are significantly different (*p* ≤ 0.05). IBW = Initial body weight; FBW = Final body weight; WGR = Weight gain rate; SGR = Specific growth rate; FIR = Feed intake rate; SR = Survival rate; PER = Protein efficiency ratio; FCR = Feed conversion ratio; CF = Condition factor; HIS = Hepatosomatic index; VSI = Viscerosomatic index; CP = Crude protein; CL = Crude lipid; CON, LLH, LMH, LHH, and HML represent different feeding regimens over 60 days divided into three phases of 20-day intervals. CON = sustained M-diet; LLH = L/L/H; LMH = L/M/H; LHH = L/H/H; HML = H/M/L.

**Table 3:** Effects of five feeding regimens at differential protein levels on serum immune markers of juvenile largemouth bass (*Micropterus salmoides*).

Item	CON	LLH	LMH	LHH	HML	SEM	*p*-Value
LZM (μg/mL)	0.21 ^b^	0.20 ^c^	0.20 ^c^	0.23 ^a^	0.23 ^a^	0.004	˂0.001
C3 (μg/mL)	118.75 ^b^	113.80 ^bc^	108.33 ^c^	133.46 ^a^	133.89 ^a^	2.978	˂0.001
C4 (μg/mL)	177.63 ^b^	164.99 ^c^	176.15 ^b^	192.59 ^a^	191.22 ^a^	3.782	˂0.001
IgM (μg/mL)	144.87 ^b^	142.20 ^b^	141.51 ^b^	168.91 ^a^	164.97 ^a^	5.208	˂0.001

Note: L = Low-protein diet; M = Medium-protein diet; H = High-protein diet. a–c Values in the same row with different superscripts are significantly different (*p* ≤ 0.05). LZM = Lysozyme; C3 = Complement component 3; C4 = Complement component 4; IgM = Immunoglobulin M. CON, LLH, LMH, LHH, and HML represent different feeding regimens over 60 days divided into three phases of 20-day intervals. CON = sustained M-diet; LLH = L/L/H; LMH = L/M/H; LHH = L/H/H; HML = H/M/L.

**Table 4:** Effects of five feeding regimens at differential protein levels on serum antioxidant capacity of juvenile largemouth bass (*Micropterus salmoides*).

Item	CON	LLH	LMH	LHH	HML	SEM	*p*-Value
CAT (U/mL)	323.38 ^d^	635.49 ^b^	607.59 ^b^	563.82 ^c^	786.29 ^a^	14.661	˂0.001
T-AOC (U/mL)	8.41 ^c^	9.30 ^b^	7.56 ^d^	10.63 ^a^	6.57 ^e^	0.307	˂0.001
MDA (nmol/mL)	2.05 ^d^	2.16 ^c^	3.71 ^a^	2.55 ^b^	2.53 ^b^	0.031	˂0.001
GSH-Px, (nmol/min/mL)	10.48 ^c^	5.04 ^e^	6.11 ^d^	19.91 ^b^	33.53 ^a^	0.407	˂0.001

Note: L = Low-protein diet; M = Medium-protein diet; H = High-protein diet. a–e Values in the same row with different superscripts are significantly different (*p* ≤ 0.05). CAT = Catalase; T-AOC = Total antioxidant capacity; MDA = Malondialdehyde; GSH-Px = Glutathione Peroxidase. CON, LLH, LMH, LHH, and HML represent different feeding regimens over 60 days divided into three phases of 20-day intervals. CON = sustained M-diet; LLH = L/L/H; LMH = L/M/H; LHH = L/H/H; HML = H/M/L.

**Table 5:** Effects of five feeding regimens at differential protein levels on intestinal enzyme activities of juvenile largemouth bass (*Micropterus salmoides*).

Item	CON	LLH	LMH	LHH	HML	SEM	*p*-Value
Trypsin (U/g)	5.98 ^b^	8.85 ^a^	3.06 ^d^	3.11 ^d^	3.90 ^c^	0.104	˂0.001
Lipase (U/g)	6.16 ^a^	0.91 ^e^	2.17 ^d^	2.83 ^c^	5.29 ^b^	0.096	˂0.001
α-Amylase (U/g)	0.52 ^c^	0.44 ^d^	0.74 ^a^	0.39 ^e^	0.54 ^b^	0.005	˂0.001

Note: L = Low-protein diet; M = Medium-protein diet; H = High-protein diet. a–e Values in the same row with different superscripts are significantly different (*p* ≤ 0.05). CON, LLH, LMH, LHH, and HML represent different feeding regimens over 60 days divided into three phases of 20-day intervals. CON = sustained M-diet; LLH = L/L/H; LMH = L/M/H; LHH = L/H/H; HML = H/M/L.

**Table 6:** Effects of five feeding regimens at differential protein levels on intestinal morphologic structure of juvenile largemouth bass (*Micropterus salmoides*).

Item	CON	LLH	LMH	LHH	HML	SEM	*p*-Value
VH (mm)	0.58	0.69	0.59	0.67	0.62	0.077	0.419
VW (mm)	0.10	0.11	0.11	0.11	0.10	0.010	0.762
MT (mm)	0.80	0.86	0.80	0.86	0.83	0.076	0.682

Note: L = Low-protein diet; M = Medium-protein diet; H = High-protein diet. VH = Villus height; VW = Villus width; MT = Muscle thickness. CON, LLH, LMH, LHH, and HML represent different feeding regimens over 60 days divided into three phases of 20-day intervals. CON = sustained M-diet; LLH = L/L/H; LMH = L/M/H; LHH = L/H/H; HML = H/M/L.

**Table 7:** Effects of five feeding regimens at differential protein levels on microbial α diversity indices of juvenile largemouth bass (*Micropterus salmoides*).

Item	CON	LLH	LMH	LHH	HML	SEM	*p*-Value
Chao1	10.18	53.98	28.43	100.67	18.67	29.022	0.106
Faith-pd	5.19	5.10	4.95	7.45	3.78	1.655	0.337
Observed-species	31.20	52.63	27.5	96.2	17.43	27.953	0.108
Pielou_e	0.39	0.41	0.27	0.37	0.41	0.155	0.885
Shannon	1.90	2.21	1.30	2.45	1.67	0.782	0.656
Simpson	0.60	0.68	0.40	0.64	0.53	0.232	0.775

Note: L = Low-protein diet; M = Medium-protein diet; H = High-protein diet. CON, LLH, LMH, LHH, and HML represent different feeding regimens over 60 days divided into three phases of 20-day intervals. CON = sustained M-diet; LLH = L/L/H; LMH = L/M/H; LHH = L/H/H; HML = H/M/L.

## Data Availability

The data that support the findings of this study have been deposited into NCBI with accession number PRJNA1447498. https://www.ncbi.nlm.nih.gov/sra/PRJNA1447498 (accessed on 2 April 2026).
